# Latent tuberculosis among household contacts of pulmonary tuberculosis cases in Nairobi, Kenya

**DOI:** 10.11604/pamj.2020.37.87.21102

**Published:** 2020-09-25

**Authors:** Susan Odera, Marianne Mureithi, Andrew Aballa, Noel Onyango, Omu Anzala, Julius Oyugi

**Affiliations:** 1 Department of Medical Microbiology, University of Nairobi, Nairobi, Kenya,; 2 KAVI-Institute of Clinical Research, University of Nairobi, Nairobi, Kenya,; 3 Department of Medical Laboratory Sciences, School of Medicine, Kenyatta University, Nairobi, Kenya,; 4 Department of Clinical Medicine and Therapeutics, University of Nairobi, Kenya,; 5 University of Nairobi Institute of Tropical and Infectious Diseases, University of Nairobi, Nairobi Kenya

**Keywords:** Latent TB infection, TB household contacts, prevalence, risk factors, Kenya

## Abstract

**Introduction:**

Household Contacts (HHCs) of Pulmonary Tuberculosis (PTB) patients have a higher risk of latent tuberculosis infection (LTBI). However, its prevalence and risk factors among adults living with PTB patients are poorly documented in Kenya.

**Objective:**

to determine the prevalence and risk factors for LTBI among adult HHCs of PTB patients in Kenya.

**Methods:**

this was an analytical cross-sectional study of HHCs of PTB patients in Nairobi, Kenya. Socio-demographic data was captured on questionnaires and blood samples drawn for Interferon gamma (IFN-γ) quantification. Univariate and multivariate analyses using the Statistical Package for Social Scientists (SPSS) was used to determine the prevalence of LTBI and risk factors at 95% Confidence Interval (CI).

**Results:**

a total of 166 PTB patients yielded 175 HHCs of whom 29.7% (52/125) were males and 70.3% (123/125) were females. A majority of HHCs [65.7% (115/175)] lived in a single-room house with the patient and [37.7% (66/175)] were in the age group 30-39-years. The overall prevalence of LTBI was 55.7%, peaking among spouses of the patients [70.0% (14/20) and the 30-39 year age group [63.5% (42/66)]. Potential risk factors for LTBI included cohabiting with a PTB patient for 8 to 12 weeks [OR = 3.6 (0.70-18.5), p = 0.107], being a spouse of the patient [OR = 2.0 (0.72-5.47), p = 0.173] and sharing a single room with the patient [OR = 1.58 (0.84 - 2.97), p = 0.158].

**Conclusion:**

the high prevalence of LTBI among adult HHCs of PTB patients in this population demonstrates the need for targeted contact-screening programs in high TB transmission settings.

## Introduction

Tuberculosis (TB) is a major health problem in sub-Saharan Africa and other developing countries including Kenya, which is ranked among the top 30 countries with the highest burden TB [1, 2]. TB is a communicable disease caused by infection with *M. tuberculosis* complex organisms, which typically spreads to new hosts via airborne droplet nuclei from patients with respiratory tuberculosis disease.

A hallmark of the natural history of tuberculosis is the diverse outcome of infection. There are three possible outcomes of exposure to an infectious TB case: the infection may be cleared by sterilizing immunity [3] as evidenced by immunological assays [4]; development of active TB and development of latent tuberculosis infection (LTBI). Studies have shown that 10% of LTB infected individuals develop active TB and because of the underlying immune deficiency, HIV-infected individuals with LTBI are at 26-fold higher risk for TB reactivation [5].

Since LTBI contributes significantly to the pool of active TB cases once reactivation occurs, its diagnosis and treatment in high-risk groups, including HHCs of PTB patients is essential for reduction and ultimately elimination of TB [2]. It is therefore important to establish the prevalence and risk factors of LTBI among HHCs of PTB patients in this population, since this data will inform policy on the need for targeted contact-screening programs.

## Methods

### Study design and setting

An analytical cross-sectional study conducted in Mbagathi District Hospital (MDH), Nairobi Kenya.

### Study population

Patients with a definitive TB diagnosis through sputum smear examination, chest X-ray, and Xpert MTB/RIF technique were recruited at the outpatient and inpatient TB wards of the hospital. HHCs were defined as adults who shared meals and rooms with the patient, and were living together. The HHCs were identified during the patient visiting hours or as they accompanied their patients to the outpatient clinic.

### Sample size and data collection

One hundred and sixty six (166) TB patients were identified and from these, 175 HHCs who agreed to participate in the study gave their informed consent and were recruited. A structured questionnaire was used to capture data on the socio-demographic characteristics of the study participants and 3 blood samples collected by venipuncture from each HHCs directly into the blood collection tubes provided in the QuantiFERON®-TB Gold In-Tube kit (QFT-GIT), (Qiagen, Germany) for Interferon-gamma release assays (IGRAs). These samples were then transported within 15 minutes to research laboratories, at College of Health Sciences, University of Nairobi for further processing.

### Interferon-gamma release assays (IGRAs)

Diagnosis of LTBI infection can be made using immunodiagnostic tests such as the Tuberculin Skin Test (TST) or Interferon Gamma (IFN- γ) Release Assays (IGRAs). Both tests are used to identify individuals with previous sensitization to mycobacterial antigens. However, the sensitivity of TST is compromised in individuals with immunosuppression, cross reactions from infection with non-tuberculous mycobacteria or BCG vaccination. IGRAs identify a memory of an adaptive immune response against mycobacterial antigens, and are more sensitive than TST. They are not affected by prior BCG vaccination and look for the body´s response to TB antigens not present in other forms of mycobacteria [6].

The QuantiFERON® - TB Gold In-Tube test that was used in this study is an IGRA test for Cell Mediated Immune (CMI) responses to peptide antigens that simulate mycobacterial proteins. These proteins, ESAT-6, CFP-10, and TB 7.7 are absent from all BCG strains and from most non-tuberculous mycobacteria with the exception of *M. kansasii, M. szulgai and M. marinum*. Individuals infected with *M. tuberculosis* complex organisms usually have lymphocytes in their blood that recognize these and other mycobacterial antigens. This recognition process involves the generation and secretion of the cytokine, IFN-γ. The detection and subsequent quantification of IFN-γ forms the basis of this test. The QuantiFERON® - TB Gold In-Tube test (QFT GIT) (Qiagen, Germany) test was performed according to the manufacturers instructions. Briefly, blood was collected by venipuncture from each HHCsinto 3 blood collection tubes one containing M. tuberculosis peptide antigens ESAT-6, CFP-10, and TB 7.7; one containing a mitogen, and one nil tube, with no antigen. The Mitogen-stimulated plasma sample served as an IFN-γ positive control for each specimen tested. Whole blood was incubated for 16 hours at 37°C and transferred to 4oC until processing, but for no longer than 48 hours. Tubes were centrifuged at 2000g for 15 min, and then supernatants were stored at 80oC until the QFT ELISA could be conducted. Supernatants and IFN-? standards (50 ml) plus conjugate (50 ml) were incubated for 2 hours, washed 6 times, and incubated for 30 min with substrate solution. After 30 min, 50 ml stop solution was added and the wells and the plates were read at 450 nm with a 650 nm reference filter. Concentrations of IFN-? were calculated based on the standard curve and test outcomes (positive, negative, or indeterminate) determined using a mathematical algorithm from the manufacture. Samples of participants with indeterminate QFT results were rerun and positive or negative outcomes on second run interpreted as their final results. Patients with indeterminate on second run were scored as QFT indeterminate. A test was positive if the ELISA value for interferon gamma (IFN-γ) was above the Nil IFN-γ value (> 0.35 IU/ml).

### Statistical analysis

Socio-demographic data were extracted from the study questionnaires and entered into a worksheet using the Statistical Package for Social Scientists (SPSS) software (version 21). Then, data were scrutinised for inconsistencies, typing errors and missing data, and the dataset cleaned following the published guidelines on data cleaning [7]. Descriptive statistics were explored and presented as tables, the prevalence of LTBI computed following the guidelines by Ward, 2013, and risk factors for LTBI determined by testing for fitness by Chi-square tests at 95% CI. p < 0.05 was significant [8].

### Ethical considerations

This data presented here was obtained in a larger study on “The role of Human Leukocyte Antigens and *M. tuberculosis* strain variation in susceptibility to infection among pulmonary tuberculosis patients in Kenya”. The Kenyatta National Hospital/University of Nairobi Ethics and Research Committee (KNH/UoN ERC) reviewed and approved the study, Reference No. KNH-ERC/A/392. This manuscript is original and is not currently under consideration by another journal.

## Results

### Socio-demographic characteristics of household contacts

Of the one hundred and seventy-five (175) HHCs recruited, 29.7% (52/175) were male and 70.3% (123/175).were female. Approximately 37% (66/175) were 30-39 years old, while 39.4% (69/175) had lived with the TB patients for over 20 weeks. About 38.9% (68/175) had secondary education, 42.9% (75/175) were unemployed, while 65.7% (115/175) lived in a one-room structure with the TB patient. Cigarette smoking, alcohol consumption, and HIV sero-positivity constituted 5.1% (5/175), 16.6% (29/175), and 9.1% (16/175) of the HHCs respectively (Table 1).

**Table 1:** socio-demographic characteristics of household contacts of TB patients, N = 175

	N	%
**Gender**		
Male	52	29.7
Female	123	70.3
**Age group**		
18-29	53	30.3
30-39	66	37.7
40-49	29	16.6
50+	27	15.4
**Relation to patient**		
Spouse	20	11.4
Others	155	88.6
**Duration of association**		
1-4 Weeks	58	33.1
4.1-8 Weeks	32	18.3
8.1-12 weeks	10	5.7
16.1-20 Weeks	6	3.4
>20.1 Weeks	69	39.4
**Educational Level**		
None	6	3.4
Primary	55	31.4
Secondary	68	38.9
College	46	26.3
Occupation		
Professional	69	39.4
House wife	31	17.7
Unemployed	75	42.9
**Room sharing**		
Yes	115	65.7
No	60	34.3
**Number of patients in household**		
1	170	97.1
>1	5	2.9
**Smoking status**		
Smoker	9	5.1
Previous smoker	4	2.3
Non smoker	162	92.6
**Alcohol use**		
Yes	29	16.6
No	146	83.4
**HIV sero-status**		
Positive	16	9.1
Negative	159	90.9

### Prevalence of LTBI among household contacts

**Figure 1:**
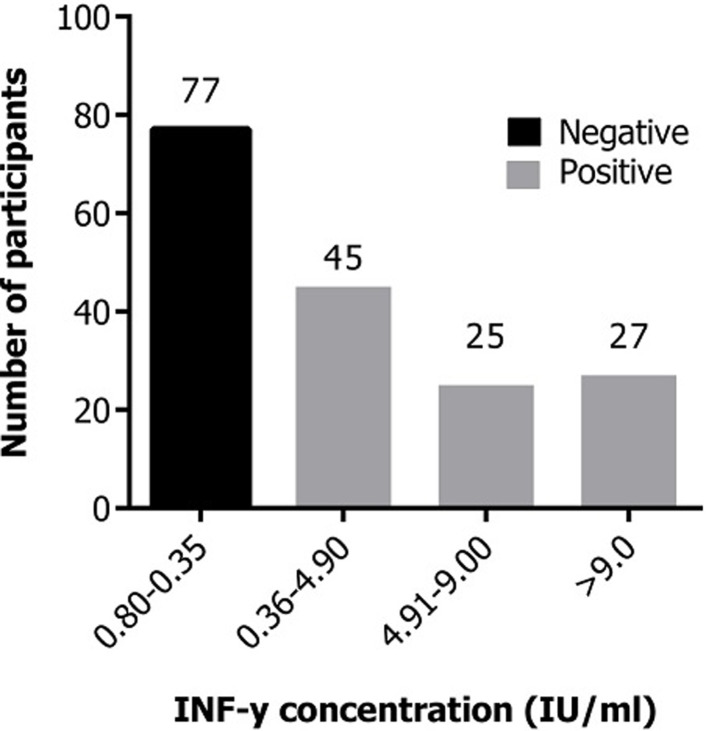
distribution of ELISA test values for the QFT-GIT test among household contacts

Of the 175 HHCs recruited only 174 had data on the QFT-GIT assay used for diagnosis of LTBI, and therefore were included in our univariate analyses. The overall prevalence of LTBI among the HHCs was found to be 55.7% (97/174). A test was positive if the ELISA value for interferon gamma (IFN-γ) using the QFT-GIT assay was above the Nil IFN-γ value (> 0.35 IU/ml). A majority of LTBI cases 46.4% (45/97) had an interferon gamma (IFN-γ) ELISA value of between 0.36 and 4.9; while 27.8% (27/97) had a value greater than 9.0 IU/ml (Figure 1). The HHCs were stratified according to socio-demographic characteristics and the prevalence of LTBI computed in each group. Analysis by gender revealed an LTBI prevalence of 58.8% (30/51) in males and 54.5% (67/123) in females. By age, LTBI exhibited a dome-shaped frequency curve, which peaked at 63.6% (42/66) among HHCs aged 30-39 years old. The 40-49-year-old group was the second most affected group with 60.7% (17/28) testing positive while 50% (11/22) of 50-59 year olds were affected. Among the HHCs who had a spousal relationship with the TB patient, the prevalence for LTBI was 70.0% (14/20) while the other relations had a prevalence of 53.9% (83/154).Analysis of the duration of association with the TB patient revealed a higher prevalence of LTBI at 80% (8/10) among HHC who were living with TB patients for 8.1-12 weeks, compared to the categories of duration of weeks of association which ranged from 50 to 56.3% prevalence (Table 2). HHCs that had no formal education had an LTBI prevalence of 66.7% (4/6) while those who had tertiary level education had a prevalence of 47.8% (22/46). Most of the HHCs lived in a single room house with the TB patient and as such had a close proximity to the TB patient. A prevalence of 59.6% (68/114) was reported amongst the HHCs who shared a room with the patient (Table 2).

**Table 2:** prevalence of LTBI and demographics of household contacts

Characteristic	N	Total	(%)
Gender			
Male	30	51	58.8
Female	67	123	54.5
Age group			
18-29	26	53	49.1
30-39	42	66	63.6
40-49	17	28	60.7
50-59	11	22	50.0
60-69	1	4	25.0
70-79	0	1	0
**Relationship to patient**			
Spouse	14	20	70.0
Others	83	154	53.9
Duration of association			
1-4 Weeks	30	57	52.6
4.1-8 Weeks	18	32	56.3
8.1-12 weeks	8	10	80.0
16.1-20 Weeks	3	6	50.0
>20.1 Weeks	38	68	55.1
**Educational Level**			
None	4	6	66.7
Primary	31	54	57.4
Secondary	40	68	58.8
College	22	46	47.8
Occupation			
Professional	37	69	53.6
House wife	18	31	58.1
Unemployed	42	74	56.8
**Room sharing with TB patients**			
Yes	68	114	59.6
No	29	60	48.3
**Number of TB patients in household**			
1	95	168	56.2
2	2	5	40.0
Smoking status			
Smoker	5	9	55.6
Previous smoker	2	4	50.0
Non smoker	90	161	55.9
Alcohol use status			
Yes	14	28	50.2
No	83	146	56.8
**HIV sero-status**			
Positive	6	16	37.5
Negative	9	158	57.6

### Risk factors for LTBI

Several risk factors for LTBI were investigated in this study. Although a slightly higher prevalence of LTBI was reported in males, the risk of LTBI did not vary significantly between males and females [OR = 1.09 (0.62 - 2.31, (p = 0.531)].].The risk of LTBI was higher among older HHCs, with the 30-39 year old age group [OR = 1.82 (0.87 - 3.79), p = 0.110] and 40-49 year old age group [OR = 1.60 (0.63 - 4.07), p = 0.317] having a higher odds of infection in reference to 18-29 year old. Finally, the odds of LTBI were higher among spouses than other household members [OR = 2.0 (0.72 - 5.47), p = 0.173], HHCs who shared rooms with TB patients [OR = 1.58 (0.84 - 2.97), p = 0.153], and HHCs who cohabited with TB patients for 8.1-12 weeks [OR = 3.60 (0.70 - 18.5), p = 0.107]. A tertiary level of education [OR = 0.46 (0.07 - 2.76), p = 0.385] was protective against infection with TB (Table 3).

**Table 3:** risk factors for LTBI among TB household contacts, N = 174

	LTBI Positive	OR	95% CI	p-value
**Gender**				
Male	30 (58.8)	1.19	0.62 - 2.31	0.531
Female	67 (54.5)	0.89	0.42 - 1.57	0.531
**Age group**				
18-29	26 (49.1)			Ref.
30-39	42 (63.6)	1.82	0.87 - 3.79	0.110
40-49	17 (60.7)	1.60	0.63 - 4.07	0.317
50+	12 (44.4)	0.83	0.32 - 2.11	0.696
**Relation to patient**				
Spouse	14 (70.0)	2.00	0.72 - 5.47	0.173
Others	83 (53.9)	0.50	0.18 - 1.37	0.173
**Duration of association**				
1-4 Weeks	30 (49.1)			Ref.
4.1-8 Weeks	18 (63.6)	1.16	0.48 - 2.76	0.742
8.1-12 weeks	8(60.7)	3.60	0.70 - 18.5	0.107
16.1-20 Weeks	3 (50.0)	0.90	0.16 - 4.84	0.902
> 20.1 Weeks	38 (25.0)	1.10	0.54 - 2.23	0.784
**Educational Level**				
None	4 (66.7)			Ref.
Primary	31 (57.4)	0.67	0.11 - 4.00	0.663
Secondary	40 (58.8)	0.71	0.12 - 4.17	0.708
College	22 (47.8)	0.46	0.07 - 2.76	0.385
Occupation				
Professional	37 (53.6)			Ref.
House wife	18 (58.1)	1.20	0.50 - 2.82	0.680
Unemployed	42 (56.8)	1.14	0.58 - 2.20	0.707
**Room sharing**				
Yes	68 (59.6)	1.58	0.84 - 2.97	0.153
No	29 (48.3)	0.63	0.33 to 1.19	0.153
**Patients in household**				
1	95 (56.2)	1.93	0.31 - 11.8	0.472
2	2 (40.0)	0.52	0.08 - 3.19	0.472
Smoking status				
Smoker	5 (55.6)			Ref.
Previous smoker	2 (50.0)	0.80	0.07 - 8.48	0.853
Non smoker	90 (55.9)	1.01	0.26 - 3.92	0.984
**Alcohol use**				
Yes	14 (50.0)	0.76	0.33 - 1.71	0.504
No	83 (56.8)	1.32	0.58 - 2.96	0.504
**HIV sero status**				
Positive	6 (37.5)	0.44	0.15 - 1.28	0.123
Negative	91 (57.6)	2.26	0.78 - 6.54	0.123

## Discussion

Tuberculosis (TB) continues to be a global concern because of its high infectivity, mortality, and cost of therapy. In addition, the patients´ families are confronted with extra social and clinical burdens associated with TB disease [9]. There is an increasing awareness of the problem LTBI poses to HHCs and therefore the need to protect such at-risk groups. In Kenya, however, data on LTBI is mainly anecdotal, which hinders its active management. To fill this gap, we established the prevalence of LTBI and its possible risk factors in a cohort of vulnerable HHCs of active TB patients who were seeking health care services in a per-urban public district hospital in the Kenyan capital, Nairobi. Overall, it was evident that LTBI is a common yet neglected problem for HHCs, with factors such as their age, relation to active TB patients, and the length of time spent cohabiting with or caring for TB patients leading lead to repeated exposure to *Mycobacterium tuberculosis* and thus the risk of developing LTBI.

We reported a high prevalence of LTBI among HHCs, with the QFT-GIT results of over half our participants indicating positivity for LTBI. For HHCs with complete data (174), the prevalence of LTBI was 55.7%, which was higher than the global average of 23% [10], and 34% in Georgia and 12.7% in Singapore [11, 12]. However, similar results have been reported in Ethiopia, where the prevalence of LTBI is observed as 63.7% [13]. The discrepancies in prevalence might be associated with the methodology used for LTBI diagnosis and the disproportional distribution of TB worldwide. Some of the prevalence studies used the TST assay for LTBI diagnosis, a test whose accuracy measure has been confounded by Bacillus Calmette - Guérin (BCG) vaccination and non-tuberculous mycobacterial (NTM) infections [14]. Data from the WHO indicating that 2,480,000 TB cases are reported in Africa every year, with 25% of these cases dying because of TB or TB complications [15]. The incidence in Africa is significantly higher than in the Americas, the East Mediterranean, and in Europe, where the TB is estimated to infect 282,000, 771,000, and 273,000 people annually [16]. The higher exposure to *M. tuberculosis* in Africa increases the risk of infection and therefore the prevalence of LTBI. People with other mycobacterial infections also often have false positive reactions to ESAT-6, CFP-10, and TB7.7 antigens, as the genes encode the proteins found in *Mycobacterium kansasii, Mycobacterium szulgai*, and *Mycobacterium marinum* [17]. Even though the prevalence of Non-Tuberculous Mycobacteria (NTM) is estimated to be approximately 5-15% in Africa [18], we did not control for NTM infections in this study.

Several known risk factors for LTBI from literature were investigated. They included gender, age group, and relationship with the TB patient, duration of association with the patient, sharing a room with the patient, education level, use of cigarettes, alcohol use, as well as the HIV sero-status. Our finding that 30 - 49year old HHC were susceptible to LTBI than younger ones (18-29 years) was consistent with the findings of in Mongolia and in India [19, 20]. In both studies, an increasing age was associated with a high risk of LTBI/TB. An age specific prevalence of LTBI among HHCs of TB patients observed an increased prevalence of infection in older children and young adults [21], and proposed the need to expand TB preventive therapy to include all HHCs. Menzies et al., 2007 reviewed article also reported a higher risk of LTBI among the elderly - a finding that was corroborated by in Ghana and in a tuberculosis-prevalent country [22, 23]. According to Zhang *et al*. 2019, elderly caregivers are more likely to spend a longer time caring for TB patients than younger ones in hospitals and homes [24]. As such, these individuals have a higher cumulative exposure to M. tuberculosis through physical contact and or social interactions, which predisposes them to a higher risk of infection, morbidity, and mortality. Moreover, because the immunity of humans diminishes with increasing age, elderly HHCs are less likely to clear the *Mycobacterium tuberculosis* infections that they are exposed to in their high TB settings, which increase the risk of persistent infection further.

The prevalence of LTBI was slightly higher among males at 58.8%than females at 54.5%. However, after our univariate analyses, gender was not identified as a risk factor for LTBI, as was the case in a cross sectional study of final year medical students in Kenya [25] and HCWs in Kigali, Rwanda [26]. In China, men were less susceptible to LTBI [27], while females had a significantly lower risk of LTBI in South Korea [28]. The inconsistence in the occurrence of LTBI by gender might be due to the variability in the structure of households in different regions and not differences in the susceptibility of the two genders to TB infections. In Kenya, most families live in rented single or one bedroom houses, where men and women share rooms [29]. In such communal settings, Menzies et al., 2007 proposes that the risk of transmission of infectious diseases such as TB might not differ by the occupation or gender of HHCs, if their risk status (such as the length of stay with active TB patients) is comparable [22]. In our study, room sharing was common (65.7%), with most HHCs cohabiting with TB patients for over five months (39.4%). A tertiary level of education seemed to be protective against LTBI. In reference to HHCs with no formal education, the odds of LTBI was 0.46 (0.07 - 2.76, p = 0.385 among HHCs with a tertiary education and 0.71(0.12 - 4.17), p = 0.71 among HHCs with secondary education. Similar results have been reported from Uganda, identifying a limited knowledge on TB among HHCs as one of the barriers of for tuberculosis contact investigation and therefore its control [30]. Gil *et al*. in 2018 [31] reported that a significant gap in knowledge about TB among HHCs increased the risk of disease transmission and reiterated the need for education campaigns at the community level, which can address misconceptions on causation and transmission of TB. The WHO recommends routine contact investigation in TB high burden countries through counseling and education of HHCs, who are the primary caregivers for active TB patients at home (WHO, 2018). Even though a majority of our respondents indicated that they had access to recent information on TB (88.6%), social support (94.3%), and counseling sessions for TB patients upon diagnosis (92%), this were not tested formally.

Immunosuppression is an independent risk factor for LTBI [32]. People with HIV have a weakened immune system and therefore are at high risk of infections such as TB. In our study, 90% (159/175) of the HHCs recruited were sero-negative. The odds of having a positive LTBI diagnosis using the QFT-GIT test was 2.26 (0.78 - 6.54) when HHCs had a HIV sero-negative status, even though the relationship was not statistically significant (p= 0.123). This discrepancy might be related to the low specificity of the QFT-GIT test in HIV seropositive patients. In the study by Legesse *et al*. 2010 in Ethiopia, the sensitivity of the QFT-GIT test among HIV seropositive patients was 83.3%, while its specificity was significantly lower at 50% [13]. The performance of QFT-GIT depends on stimulation of CD4+ T-cells, which limits its performance in HIV-positive individuals, especially if they have a reduced capacity for IFN-gamma secretion from CD4+ T-cells. As such, because the QFT-GIT in-tube kit would most likely perform better in people with a negative HIV sero-status, interpretation of results in the absence of sero-status data would be limited. A newer generation of the QFT assay - the QuantiFERON®-TB Gold Plus, has been developed to overcome such limitations. In addition to the antigens found in the QFT-GIT kit, the QFT-Gold plus has shorter peptides of the same antigens, which simulate CD8+ T-cells to improve its sensitivity in HIV seropositive patients. The 2018 WHO guidelines included a new recommendation on the use of TST or IGRA to test for LTBI [2].

Medical management of an individual who tests positive using the IGRA test would involve an evaluation of epidemiologic and medical history and other clinical information. If the individual is at risk for progression to active TB and has signs and symptoms suggestive of active disease, additional evaluation would be required. Treatment of selected persons with LTBI using preventive therapy aims at preventing active disease. The 2018 WHO Guidelines included policy documents for programmatic management of LTBI in people living with HIV and household contacts of TB patients and other at-risk groups. As highlighted in these documents, the cascade of care for managing LTBI would include identification of at-risk populations, ruling out active TB disease, testing for LTBI, providing treatment, monitoring adverse events, adherence and completion of treatment [15]. One of the identified at risk groups are household contacts of people with bacteriologically confirmed pulmonary TB. A study done in Ethiopia showed that individuals that had a TB household member had an increased risk of developing TB by 3-fold [33]. In such groups it is necessary to identify the intensity of exposure, the risk for development of active TB and ascertainment of infection through testing for LTBI.

Kenya is recognized as one of the high burden TB countries. Contact investigation as outlined in WHO guidelines has not yet been fully implemented by the National Tuberculosis program in Kenya. The TB program in Kenya recommends Isoniazid Preventive Therapy administration to household contacts of PTB patients who are either less than 5 years old or are HIV infected adults [34]. The current national guidelines should be expanded to implement the standardized contact investigation that would include active screening, evaluation and considered chemoprophylaxis of HHCs of PTB patients, including HIV negative adults.

## Conclusion

The high prevalence of LTBI among HHCs of PTB patients in this population demonstrates the need for targeted contact-screening programs in high TB transmission settings. This would enable early detection of LTBI and initiation of treatment of at-risk groups such as HHCs. Investigations should be targeted on close contacts of PTB patients such as spouses, elderly HHCs, and HHCs who share rooms with PTB patients for weeks. Efforts should also focus on airborne infection control measures in homes and on sensitizing at-risk HHCs on LTBI and the importance of preventive treatment to avoid reactivation.

### What is known about this topic

- Close contacts of pulmonary tuberculosis patients are at a high risk of infection because the highly infectious etiologic agent Mycobacterium tuberculosis is spread airborne;
- Individuals who have LTBI are not infectious and do not show any clinical symptoms but are important reservoirs for disease reactivation;
- The diagnosis and treatment of LTBI in high-risk groups is essential for reduction and ultimately elimination of TB.

### What this study adds

- The high prevalence of LTBI in adult household contacts of TB patients emphasizes the need for TB programs to invest more in the screening and treatment of household contacts in high transmission settings;
- TB programs in resource limited settings should target LTBI screening of spouses of TB patients and HHCs residing in a one-room structure with the patient;
- TB prevention efforts should focus on sensitizing household contacts on the importance of preventive treatment to avoid reactivation.
